# Complete chloroplast genome of *Cardamine hupingshanensis* K.M.Liu, L.B.Chen, H.F.Bai & L.H.Liu (Brassicaceae) in Enshi, Hubei

**DOI:** 10.1080/23802359.2022.2113752

**Published:** 2022-08-29

**Authors:** Xiuqing Liu, Siying Zhang

**Affiliations:** a Forestry College, Xinyang Agriculture and Forestry University, Xinyang, China; b Tourism Management College, Xinyang Agriculture and Forestry University, Xinyang, China

**Keywords:** Brassicaceae, *Cardamine hupingshanensis*, chloroplast genome, phylogeny

## Abstract

*Cardamine hupingshanensis* K.M.Liu, L.B.Chen, H.F.Bai & L.H.Liu 2008, also called *Cardamine enshiensis*, belongs to the genus *Cardamine*, Brassicaceae. As a plant with selenium enrichment ability, it has high development value. Here, we analyzed the chloroplast genome of *C. hupingshanensis*. The complete chloroplast genome had a total size of 154,832 bp with a typical quadripartite structure, including a large single-copy region (LSC, 83,908 bp) and a small single-copy region (SSC, 17,938 bp), separated by a pair of inverted repeat regions (IRs, 26,493 bp). Genome annotation showed the chloroplast genome contained 113 unique genes, including 79 protein-coding genes, 30 tRNA genes, and four rRNA genes. A total of 143 SSRs were found in the chloroplast genome. Phylogenetic analysis showed that *C. hupingshanensis* was closer to the *C. circaeoides* and *C. lyrata*. This chloroplast genome resource will be useful for study of the phylogeny and evolution of *Cardamine* in the future.

*Cardamine hupingshanensis* K.M.Liu, L.B.Chen, H.F.Bai & L.H.Liu 2008, also called *Cardamine enshiensis*, belongs to the Brassicaceae family, is a selenium (Se) hyperaccumulator plant in Enshi, Hubei Province, China (Shao et al. 2007; Bai et al. 2008; Cui et al. 2018). Selenium (Se) is an essential trace element for human and animals, which is necessary for the synthesis of selenoprotein and selenoenzymes (Butler et al. 1989; Behne and Kyriakopoulos 2001; Rafferty et al. 2003). For humans and animals, plants are the main source of selenium intake, so adequate selenium in plants is very important for health (Sargent 2008; Williams et al. 2009). Some previous studies showed that some Cabombaceae, Liliaceae, and Fabaceae plants had high ability to accumulate Se (Sharma et al. 2010; Freeman and Bañuelos 2011), such as the *Astragalus bisulcatus* (Pilon-Smits et al. 2009) and *Stanleya pinnata* (Freeman et al. 2006). In recent years, the researchers systematically analyzed the whole genome of *C. hupingshanensis*, and studied the mechanism underlying Se tolerance and hyperaccumulation (Huang et al. 2021). Nevertheless, the phylogeny and evolution of the chloroplast genome still need to be explored for *C. hupingshanensis*. Hence, we characterized the chloroplast genome of *C. hupingshanensis* that may contribute to understanding the phylogenetic relationship of *Cardamine*.

The material of *C. hupingshanensis* was collected from Tiechangba, Enshi, Hubei in China (110°08′43″E, 30°47′22″N, and altitude 1150 m). A specimen was deposited in the herbarium of the Forestry College, Xinyang Agriculture and Forestry University (voucher number: ENS001, https://www.xyafu.edu.cn/lxy/, Zhang Siying, 2019300002@xyafu.edu.cn). The total genomic DNA of leaves was extracted using modified CTAB method (Doyle and Doyle 1987; Chen et al. 2014). Genomic DNA was sequenced using the Illumina NovaSeq platform of Biomarker Technologies Company (Beijing, China). A total of 3.3 Gb raw reads were generated, and low-quality sequences were filtered by using Trimmomatic v. 0.39 (Bolger et al. 2014) to obtain clean data. The clean data were quality-controlled by using FastQC v. 0.11.9 (Simon 2020). Then, the complete chloroplast genome was assembled by using GetOrganelle v. 1.7.5 (Jin et al. 2020), the parameters set as follows: -R = 15, -F = embplant_pt, -k = 21, 45, 65, 85, and 105. We used Bandage v. 0.8.1 (Wick et al. 2015) to check the integrity of the assembly result, and then used CPGAVAS2 (Shi et al. 2019) and PGA (Qu et al. 2019) for genome annotation. Finally, the complete chloroplast genome had a total size of 154,832 bp (ON322745) with a typical quadripartite structure, including a large single-copy region (LSC, 83,908 bp) and a small single-copy region (SSC, 17,938 bp), separated by a pair of inverted repeat regions (IRs, 26,493 bp). The total GC content of the chloroplast genome was 36.27%. The chloroplast genome encoded 113 unique genes, including 79 protein-coding genes, 30 tRNA genes, and four rRNA genes. MIcroSAtellite software (Beier et al. 2017) was used for simple repeat sequence (SSR) analysis in chloroplast genome of *C. hupingshanensis*, the number of repetitions was set to 10, 6, 5, 5, 5, and 5 from mononucleotide to hexanucleotide, respectively. The simple sequence repeat analysis showed that 143 SSRs were identified in the chloroplast genome of *C. hupingshanensis*. Moreover, the genome size, GC content, gene number, and gene order were similar to other *Cardamine* chloroplast genomes.

**Figure 1.**
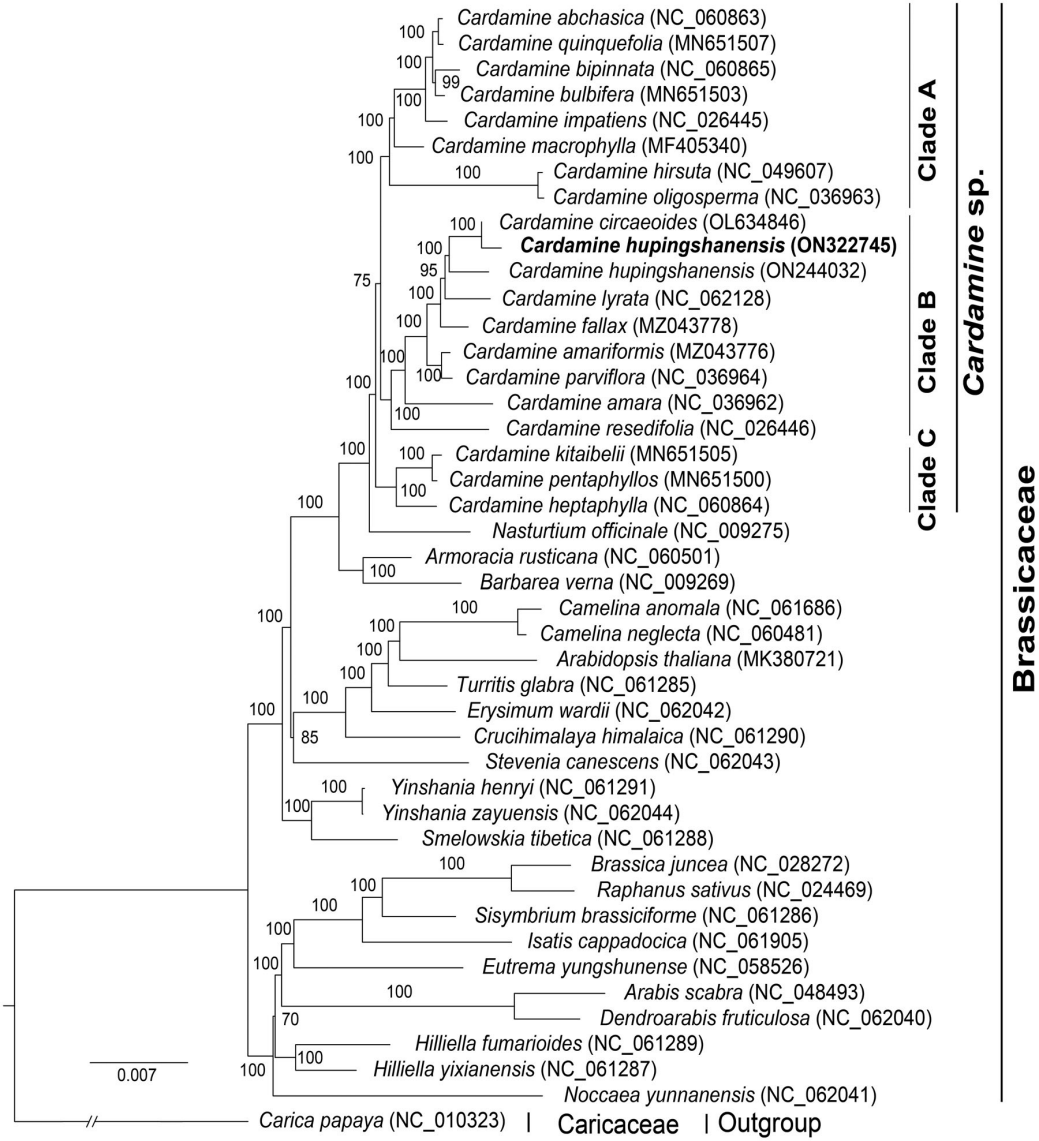
Phylogenetic tree reconstructed by maximum-likelihood (ML) based on the 79 protein-coding genes of chloroplast genome.

In order to explore the phylogenetic relationship of *C. hupingshanensis*, the complete chloroplast genomes of 42 species from Brassicaceae and one species from Caricaceae were obtained from the GenBank database. We used the PhyloSuite v. 1.2.2 (Zhang et al. 2020) to extract 79 protein-coding genes from the chloroplast genome annotation files. Each protein-coding gene sequence was aligned by using MAFFT v. 7.4 (Katoh and Standley 2013), and then 79 aligned sequences were concatenated by using PhyloSuite v. 1.2.2 (Zhang et al. 2020). With the *Carica papaya* as the outgroup, the phylogenetic tree (Figure 1) was constructed by maximum-likelihood (ML) method with IQ-TREE v. 2.1.2 (Nguyen et al. 2015) under the optimal model of GTR + F + R4. The bootstrap value was 1000. The analysis result showed that *Cardamine* and *Nasturtium* were closer, and were monophyletic groups to each other. There were three main branches in the genus *Cardamine*. Clade A included *C. abchasica*, *C. quinquefolia*, *C. bipinnata*, *C. bulbifera*, *C. impatiens*, *C. macrophylla*, *C. hirsuta*, and *C. oligosperma*. Clade B included *C. circaeoides*, *C. hupingshanensis*, *C. lyrate*, *C. fallax*, *C. amariformis*, *C. parviflora*, *C. amara*, and *C. resedifolia*. Clade C included *C. kitaibelii*, *C. pentaphyllos*, and *C. heptaphylla*. In the genus *Cardamine*, *C. hupingshanensis* was closer to *C. circaeoides* and *C. lyrata*. This finding was similar to previous research results (Hu et al. 2015; Raman and Park 2021, 2022; Raman et al. 2021; Xu et al. 2022). Our results provide valuable data and shed light on the phylogenomic study of *Cardamine* and Brassicaceae.

## Ethical approval

In this study, all experimental protocols relating to plant experiments were in accordance with the measures for the Wild Plant Protection Regulations of Henan Province (approved by the Henan Provincial Government in 2007) and Plant Protection Regulations of Hubei Province, China (approved by the Hubei Provincial Government in 2009). The Plant Herbarium of Xinyang Agriculture and Forestry University approved the collection and research of this material. All the research meets ethical guidelines and adheres to the legal requirements of the study country.

## Author contributions

Zhang Siying conceptualized and designed research; Liu Xiuqing analyzed data and wrote the manuscript. All authors read and approved the final manuscript.

## Data Availability

The genome sequence data that support the findings of this study are openly available in GenBank of NCBI at https://www.ncbi.nlm.nih.gov under the accession no. ON322745. The associated ‘BioProject’, ‘Bio-Sample’, and ‘SRA’ numbers are PRJNA830645, SAMN27735482, and SRR18884471, respectively.
